# Supplementary material for: A Comprehensive Review of the Effects of Glycemic Carbohydrates on the Neurocognitive Functions Based on Gut Microenvironment Regulation and Glycemic Fluctuation Control
Source: Nutrients. 2023 Dec 12;15(24):5080. doi: 10.3390/nu15245080 (PMC10745758; doi:10.3390/nu15245080)
Supplement: Supplementary file 1 [file nutrients-15-05080-s001.zip › nutrients-2748287-supplementary.pdf]

Table S1. Effects of monosaccharide and disaccharide intake on gut microbiota

Sugar	Fermentation	Gut microbiota a)		Other metabolites	Ref
		Abundance up	Abundance down		
Glucose	Mice/<br>In vivo	**Genus**:<br>*Enterobacteria*	**Genus**:<br>*Lactobacillus*	**Fecal:** acetate↑, aspartate↓, formate↑, methanol↑, propionate↑, pyruvate↑	[1]
	Mice/<br>In vivo b)	**Phylum:** *Firmicutes, Verrucomicrobia, Proteobacteria*	**Phylum:**<br>*Deferribacteres, Bacteroidetes*	**Serum:** total cholesterol↑, low-density lipoprotein (LDL)-cholesterol↑, endotoxin↑	[2]
Fructose	Mice/<br>In vivo	**Phylum:** *Firmicutes, Verrucomicrobia, Proteobacteria, Deferribacteres*<br>**Family**: *Lachnospiraceae Ruminococcaceae*	**Phylum:** *Bacteroidetes*<br>**Family**: *Deferribacteraceae, Helicobacteraceae*	**Fecal:** total SCFAs↓, Acetate↓, propionate↓, butyrate↓<br>**Serum:** endotoxin↑	[3]
	Mice/<br>In vivo	**Genus**:<br>*Enterobacteria*	**Genus**:<br>*Lactobacillus*	**Fecal:** acetate↑, formate↑, aspartate↓, butyrate↓, xylose↑, pyruvate↑, glutamate↓, methanol↑, succinate↑, taurine↑	[1]
Sucrose	Rats/<br>In vivo	**Phylum:** *Bacteroidetes*<br>**Family**: *Bacteroidaceae*, *Erysipelotricgaceae*, *Turicibacteraceae*	**Phylum:** *Firmicutes*<br>**Family**: *Ruminococcaceae, Clostridiales Lactobacillacae*	—	[4]
	Mice/<br>In vivo	**Phylum:** *Firmicutes, Proteobacteria*<br>**Genus**: *Alistipes*, *Bacteroides*	**Phylum:** *Bacteroidetes*<br>**Genus:** *Lactobacillus, Alloprevotella, Akkermansia*	—	[5]
	Mice/<br>In vivo	**Order:** *Clostridiales*, *Erysipelotrichales Lactobacillales*	**Order:** *Bacteroidales*<br>**Genus**: *Alistipes*, *Ruminococcus*,	—	[6]

			Butyricicoccus		
	Mice/<br>In vivo	**Phylum:** *Verrucomicrobia*<br>**Family**: *Verrucomicrobiaceae*<br>*Porphyromonadaceae*	**Phylum:** *Firmicutes, Tenericutes*<br>*Proteobacteria*<br>**Family**: *Prevotellaceae*<br>*Lachnospiraceae, Anaeroplasmataceae*	**Fecal:** total SCFA↓<br>acetate↓, butyrate↓	[7]
Sucrose mimics c)	Rats/<br>In vivo	**Phylum:** *Firmicutes, Proteobacteria*<br>**Genus**: *Sutterella Parabacteroides,*<br>*Bacteroides, Lactobacillus, Oscillosoira*	**Phylum:** *Verrucomicrobia, Bacteroidetes*<br>**Genus**: *Prevotella, Akkernansia,*<br>*Paraprevotella, Mucisoirillum*	—	[8]
	Rats/<br>In vivo	**Family**: *Rikenellaceae, Bacteroidaceae*<br>*Enterobacteriaceae, Bifidobacteriaceae*<br>*Clostridiaceae.1, Lactobacillaceae*<br>**Genus**: *Bacteroides, Clostridium sensu*<br>*stricto, Alistipes, Lactobacillus,*<br>*Parasutterella, Bifidobacteriaceae,*	**Family**:<br>*Prevotellaceae, Ruminococcaceae*<br>*Lachnospiraceae*<br>**Genus**:<br>*Prevotella*,<br>*Lachnospiracea incertae sedis*	—	[9]

Notes:

a) This column only lists the level of discussion highlighted in the references.

b) In this study, both high glucose/fructose diets were fed, both of which caused the same trend of change compared to normal diet.

c) In some references, glucose and fructose are mixed in certain proportions, and such "mixed sugars" are classified as "sucrose mimics" in this table.

—: Not tested; ↑: increased; ↓: decreased.

Table S2. Effects of starch with different digestive properties intake on gut microbiota

Type	Starch origin	Fermentation	Gut microbiota		SCFA	Ref
			Abundance up	Abundance down		
RDS	Maltodextrin	Mice/ In vivo	cecal bacterial loads↑ chronic inflammation↑ disease susceptibility↑ gut microbes dysbiosis suppressed intestinal anti-microbial defense mechanisms			[10,11]
	Pregelatinized maize starch	Mice/ In vivo	**Phylum:** *Proteobacteria, Firmicutes* **Genus:** *Alistipes, Oscillibacter, Ruminiclostridium-9*	**Phylum:** *Bacteroidetes* **Genus:** *bacteroides, Streptococcus*	isovalerate↓ valerate↓	[12]
SDS	Starch yields modified with 1,4-α-glucan branching enzyme	Mice/ In vivo	**Phylum:** *Verrucomicrobia* **Genus:** *Lactobacillus, Lachnodpiraceae, Akkermamsia, Mucispirillum, Bifidobacterium*	**Phylum:** *Proteobacteria* **Genus:** *Enterococcus, Alistipes, Odoribacter, Ruminiclostridium*	acetate↑ butyrate↑ valerate↑	[12]
	Starch-entrapped microspheres	Mice/ In vivo	**Phylum:** *Firmicutes*	**Phylum:** *Bacteroidetes*	total SCFAs↑ acetate↓ butyrate↓	[13]
			(compared with RSII, raw potato starch)			
RMD a)	Cassava starch modified with α-amylase and branching enzyme	Human Feces/ In vitro	**Genus:** *Bifidobacteria, Lactobacillus*	**Genus:** *Clostridia, Bacteroids*	Acetic↑ Butyric↑	[14]
			(compared with glucose)			
	Resistant maltodextrin	Mice/ In vivo	**Phylum:** *Firmicutes, Proteobacteria, Actinobacteria*	**Phylum:** *Bacteroidetes* **Genus:** *Bacteroides,*	—	[15]

			**Genus:** *Lactobacillus, Bifidobacterium*	*Oscillibacter, Alistipes, Prevotella, Prevotellaceae Ruminococcaceae, Ruminococcus*		
**RSI**	Isolated intact cotyledon cells from pinto bean seeds	Human Feces/ In vitro	**Phylum:** *Firmicutes* **Family**: *Lachnospiraceae, Ruminococcaceae,* ***Genus:*** *Clostridiales*	**Phylum:** *Bacteroidetes* **Family**: *Faecalibacterium* **Genus:** *Megamonas*	total SCFAs↓ acetate↓ pyruvate↓ butyrate↓	[16]
	Intact cotyledon cells isolated from red kidney beans	human intestinal microbial ecosystem	**Phylum:** *Actinobacteria* **Family**: *Coriobacteriaceae, Bifidobacteriaceae*	**Phylum:** *Firmicutes*, *Bacteroidetes* **Family**: *Bacteroidaceae, Selenomonadaceae*	butyrate↑	[17]
			(compared with microbiota taken before the application of bean treatment)			
**RSII**	High-amylose maize starch	Mice/ In vivo	**Phylum:** *Verrucomicrobia, Bacteroidetes, Actinobacteria* **Genus:** *Bifidobacterium, Akkermansia, Allobaculum*	**Phylum:** *Firmicutes* **Genus:** *Butyrivibrio, Turicibacter*	—	[18]
	Highly resistant starch rice	Feces/ In vitro	**Phylum:** *Bacteroidetes* **Genus:** *Prevotellacea Faecalibacterium*	**Phylum:** *Firmicutes, Proteobacteria* **Genus:** *Escherichia, Megamonas*	Acetate↑ propionate↑ butyrate↑ isobutyric↓ isovaleric↓	[19]
	High amylose maize starch	Human/ In vivo	**Phylum:** *Bacteroidetes, Actinobacteria* **Family:** *Ruminococcaceae, Porphyromonadaceae*	**Phylum:** *Firmicutes* **Family:** *Erysipelotrichaceae* **Genus:** *Faecalibacterium,*	—	[20]

			**Genus:** *Parabacteroides, Bifidobacterium*	*Dorea*		
	Buckwheat-resistant starch	Mice/<br>In vivo	**Phylum:** *Bacteroidetes*<br>**Genus:** *Bacteroides, Ruminiclostridium, Blautia*	**Phylum:** *Firmicutes*, *Proteobacteria*<br>**Genus:** *Turicibacter, Erysipelatoclostridium, Escherichia-Shigella*	Propionate↑<br>Butyrate↑	[21]
**RSIII**	Lotus seed resistant starch	Mice/<br>In vivo	**Family:** *Lachnospiraceae, Clostridium, Ruminococcaceae*<br>**Genus:** *Lactobacillus, Bifidobacterium*	**Family:** *Rikenellaceae, Porphyromonadaceae*	Formic acid↑<br>Acetic acid↑<br>Propionic acid↓<br>Butyrate↑	[22]
	Buckwheat-resistant starch	Mice/<br>In vivo	**Genus:** *Lactobacillus, Bifidobacterium, Enterococcus*	**Genus:** *Escherichia Coli*	Acetic acid↑<br>Butyrate↑<br>Propionic acid↑	[23]
	Retrograded tapioca starch	Young pigs/<br>In vivo	**Family:** *Strptococcaceae, Bacteroidales*<br>**Family:** *Ruminococcaceae*	**Family:**<br>*Lachnospiraceae*	—	[24]
**RSIV**	Chemically modified phosphorylated cross-linked RSIV	Human/<br>In vivo	**Phylum:** *Actinobacteria, Bacteroidetes*<br>**Genus:** *Veillonellaceae*, *Lachnospiraceae, Erysipelotrichaceae*	**Phylum:** *Firmicutes*<br>**Family:** *Ruminococcaceae*	—	[20]
		Human Feces/<br>In vitro	**Genus:**<br>*Clostridiales, Bacteroides Ruminococcus*<br>(compared with high-amylose maize starch, which is the raw material of RSIV)	**Genus:** *Blautia, Roseburia, Faecalibacterium*	total SCFAs↓<br>acetate↓<br>propionic↓ butyrate↓	[25]
		Human/<br>In vivo	**Species:** *Ruminococcus lactaris, Bacteroides acidifaciens, Blautia glucerasea, Christensenella minuta, Eubacterium*	**Species:**<br>*Enterococcus casseliflavus, Streptococcus cristatus*	butyric↑<br>propionic↑<br>valeric↑	[26]

			oxidoreducens, Parabacteroides distasonis		isovaleric↓	
**RSV**	Debranched high-amylose starch and palmitic acid	Human Feces/<br>In vitro	**Phylum:**<br>*Actinobacteria, Firmicutes*<br>**Genus:** *Blautia, Anaerostipes, Holdemanella, Bifidobacterium, Streptococcus, Collinsella, Dialister, Megamonas, Faecalibacterium*	**Phylum:** *Proteobacteria, Bacteroidetes*<br>**Genus:** *Parasutterella, Alistipes, Barnesiella, Clostridiales, Bilophila, Parabacteroides, Sutterella, Dorea, Lachnoclostridium, Flavonifractor, Phascolarctobacterium*	acetic acid↑<br>butyric acid↑<br>lactic acid↑<br>valeric acid↓	[27]
			(compared with high-amylose maize starch, which is the raw material of RSII)			
	High-amylose maize starches complexed with saturated fatty acids	Human Feces/<br>In vitro	**Phylum:** *Bacterioidetes*<br>**Genus:** *Prevotella, Megamonas, Bacteroides*	**Phylum:** *Firmicutes*<br>**Genus:** *Roseburia, Lachnospiraceae, Ruminococcus*	total SCFAs↑<br>acetate↑<br>butyrate↑<br>propionate↑	[28]
			(compared with high-amylose maize starch, which is the raw material of RSII)			

Notes:

a) RMD: Resistant maltodextrin

Unless otherwise noted, any upward or downward adjustments that appear in the table are compared to the normal diet or normal corn starch.

—: Not tested; ↑: increased; ↓: decreased.

References

1. Silva, J.; Marta, M.; Martins, F.O.; Celia, N.; Teresa, G.; Tatiana, C.; Joana, P.; Daniela, D.; Barros, A.S.; Jones, J.G. Intestinal microbial and metabolic profiling of mice fed with high-glucose and high-fructose diets. *Journal of Proteome Research* **2018**, *29*, 1703079, doi:10.1021/acs.jproteome.8b00354.
2. Do, M.H.; Lee, E.; Oh, M.-J.; Kim, Y.; Park, H.-Y. High-glucose or-fructose diet cause changes of the gut microbiota and metabolic disorders in mice without body weight change. *Nutrients* **2018**, *10*, 761, doi:10.3390/nu10060761.
3. Li, J.-M.; Yu, R.; Zhang, L.-P.; Wen, S.-Y.; Wang, S.-J.; Zhang, X.-Y.; Xu, Q.; Kong, L.-D. Dietary fructose-induced gut dysbiosis promotes mouse hippocampal neuroinflammation: a benefit of short-chain fatty acids. *Microbiome* **2019**, *7*, 1-14, doi:10.1186/s40168-019-0713-7.
4. Sun, S.; Araki, Y.; Hanzawa, F.; Umeki, M.; Oda, H. High sucrose diet-induced dysbiosis of gut microbiota promotes fatty liver and hyperlipidemia in rats. *The Journal of Nutritional Biochemistry* **2021**, *93*, 108621, doi:10.1016/j.jnutbio.2021.108621.
5. Cheng, K.; Gao, R.; Yan, X.; Huang, L.; Qin, H. Probiotics improve gut microbiota dysbiosis in obese mice fed a high-fat or high-sucrose diet. *Nutrition* **2018**, *60*, doi:10.1016/j.nut.2018.10.002.
6. Magnusson, K.R.; Hauck, L.; Jeffrey, B.M.; Elias, V.; Humphrey, A.; Nath, R.; Perrone, A.; Bermudez, L.E. Relationships between diet-related changes in the gut microbiome and cognitive flexibility. *Neuroscience* **2015**, *300*, 128-140, doi:10.1016/j.neuroscience.2015.05.016.
7. Laffin, M.; Fedorak, R.; Zalasky, A.; Park, H.; Madsen, K.L. A high-sugar diet rapidly enhances susceptibility to colitis via depletion of luminal short-chain fatty acids in mice. *Scientific Reports* **2019**, *9*, 12294, doi:10.1038/s41598-019-48749-2.
8. Noble, E.E.; Olson, C.A.; Davis, E.; Tsan, L.; Chen, Y.-W.; Schade, R.; Liu, C.; Suarez, A.; Jones, R.B.; de La Serre, C. Gut microbial taxa elevated by dietary sugar disrupt memory function. *Translational psychiatry* **2021**, *11*, 1-16, doi:10.1038/s41398-021-01309-7.
9. Noble, E.E.; Hsu, T.M.; Jones, R.B.; Fodor, A.A.; Kanoski, S.E. Early-Life Sugar Consumption Affects the Rat Microbiome Independently of Obesity. *Journal of Nutrition* **2017**, *147*, 20-28, doi:10.3945/jn.116.238816.
10. Nickerson, K.P.; Homer, C.R.; Kessler, S.P.; Dixon, L.J.; Kabi, A.; Gordon, I.O.; Johnson, E.E.; de la Motte, C.A.; McDonald, C. The dietary polysaccharide maltodextrin promotes Salmonella survival and mucosal colonization in mice. *PLoS One* **2014**, *9*, e101789, doi:10.1371/journal.pone.0101789.
11. Nickerson, K.P.; Mcdonald, C. Crohn's Disease-Associated Adherent-Invasive Escherichia coli Adhesion Is Enhanced by Exposure to the

Ubiquitous Dietary Polysaccharide Maltodextrin. *PloS One* **2012**, *7*, e52132, doi:10.1371/journal.pone.0052132.
12. Kong, H. Novel Short-Clustered Maltodextrin as a Dietary Starch Substitute Attenuates Metabolic Dysregulation and Restructures Gut Microbiota in db/db Mice. *Journal of Agricultural and Food Chemistry* **2020**, *68*, 44, doi:10.1021/acs.jafc.0c05798.
13. Kaur, A.; Chen, T.; Green, S.J.; Mutlu, E.; Martin, B.; Rumpagaporn, P.; Patterson, J.A.; Keshavarzian, A.; Hamaker, B.R. Physical Inaccessibility of a Resistant Starch Shifts Mouse Gut Microbiota to Butyrogenic Firmicutes. *Molecular Nutrition & Food Research* **2019**, *63*, 1801012, doi:10.1002/mnfr.201801012.
14. Sorndec H , W.; Rodtong, S.; Blennow, A.; Tongta, S. Impact of Resistant Maltodextrins and Resistant Starch on Human Gut Microbiota and Organic Acids Production. *Starch - Starke* **2019**, *71*, 1800231, doi:10.1002/star.201800231.
15. He, B.; Nohara, K.; Ajami, N.J.; Michalek, R.D.; Tian, X.; Wong, M.; Losee-Olson, S.H.; Petrosino, J.F.; Yoo, S.-H.; Shimomura, K. Transmissible microbial and metabolomic remodeling by soluble dietary fiber improves metabolic homeostasis. *Scientific reports* **2015**, *5*, 1-12, doi:10.1038/srep10604.
16. Guan, N.; He, X.; Wang, S.; Liu, F.; Huang, Q.; Fu, X.; Chen, T.; Zhang, B. Cell wall integrity of pulse modulates the in vitro fecal fermentation rate and microbiota composition. *Journal of agricultural and food chemistry* **2020**, *68*, 1091-1100, doi:10.1021/acs.jafc.9b06094.
17. Rovalino-Córdova, A.M.; Fogliano, V.; Capuano, E. Effect of bean structure on microbiota utilization of plant nutrients: An in-vitro study using the simulator of the human intestinal microbial ecosystem (SHIME®). *Journal of Functional Foods* **2020**, *73*, 104087, doi:10.1016/j.jff.2020.104087.
18. Sybille, T.; June, Z.; Michael, K.; Roy, M.; Maria L, M. The intestinal microbiota in aged mice is modulated by dietary resistant starch and correlated with improvements in host responses. *FEMS microbiology ecology* **2013**, *83*, 299-309, doi:10.1111/j.1574-6941.2012.01475.x.
19. Li, Z.T.; Hu, G.A.; Zhu, L.; Zhao, Z.C.; Zhan, X.B. In vitro digestion and fecal fermentation of highly resistant starch rice and its effect on the gut microbiota. *Food Chemistry* **2021**, *361*, 130095, doi:10.1016/j.foodchem.2021.130095.
20. Inés, M.; Jaehyoung, K.; Duffy, P.R.; Schlegel, V.L.; Jens, W.; Heimesaat, M.M. Resistant Starches Types 2 and 4 Have Differential Effects on the Composition of the Fecal Microbiota in Human Subjects. *Plos One* **2010**, *5*, e15046, doi:/10.1371/journal.pone.0015046.
21. Zhou, Y.; Wei, Y.; Yan, B.; Zhao, S.; Zhou, X. Regulation of tartary buckwheat - resistant starch on intestinal microflora in mice fed with high - fat diet. *Food science & nutrition* **2020**, *8*, 3243-3251, doi:10.1002/fsn3.1601.

22. Zeng, H.; Huang, C.; Lin, S.; Zheng, M.; Chen, C.; Zheng, B.; Zhang, Y. Lotus seed resistant starch regulates gut microbiota and increases short-chain fatty acids production and mineral absorption in mice. *Journal of agricultural and food chemistry* **2017**, *65*, 9217-9225, doi:10.1021/acs.jafc.7b02860.
23. Zhou, Y.; Zhao, S.; Jiang, Y.; Wei, Y.; Zhou, X. Regulatory function of buckwheat ‐ resistant starch supplementation on lipid profile and gut microbiota in mice fed with a high ‐ fat diet. *Journal of food science* **2019**, *84*, 2674-2681, doi:10.1111/1750-3841.14747.
24. Umu, Ö.C.; Frank, J.A.; Fangel, J.U.; Oostindjer, M.; da Silva, C.S.; Bolhuis, E.J.; Bosch, G.; Willats, W.G.; Pope, P.B.; Diep, D.B. Resistant starch diet induces change in the swine microbiome and a predominance of beneficial bacterial populations. *Microbiome* **2015**, *3*, 1-15, doi:10.1186/s40168-015-0078-5.
25. Wang, S.; Zhang, B.; Chen, T.; Li, C.; Huang, Q. Chemical Cross-Linking Controls in Vitro Fecal Fermentation Rate of High-Amylose Maize Starches and Regulates Gut Microbiota Composition. *Journal of Agricultural and Food Chemistry* **2019**, *67*, 13728-13736, doi:10.1021/acs.jafc.9b04410.
26. Upadhyaya, B.; Mccormack, L.; Fardin-Kia, A.R.; Juenemann, R.; Dey, M. Impact of dietary resistant starch type 4 on human gut microbiota and immunometabolic functions. *Scientific Reports* **2016**, *6*, 28797, doi:10.1038/srep28797.
27. Qin, R.; Wang, J.; Chao, C.; Yu, J.; Wang, S. RS5 Produced More Butyric Acid through Regulating the Microbial Community of Human Gut Microbiota. *Journal of Agricultural and Food Chemistry* **2021**, *69*, 3209-3218, doi:10.1021/acs.jafc.0c08187.
28. Zhou, Q.; Fu, X.; Dhital, S.; Zhai, H.; Zhang, B. In vitro fecal fermentation outcomes of starch-lipid complexes depend on starch assembles more than lipid type. *Food Hydrocolloids* **2021**, 10.1016/j.foodhyd.2021.106941, 106941, doi:10.1016/j.foodhyd.2021.106941.
